# Cartilage Targets of Knee Osteoarthritis Shared by Both Genders

**DOI:** 10.3390/ijms22020569

**Published:** 2021-01-08

**Authors:** Chenshuang Li, Zhong Zheng

**Affiliations:** 1 Department of Orthodontics, School of Dental Medicine, University of Pennsylvania, Philadelphia, PA 19104, USA; lichens@upenn.edu; 2 Division of Growth and Development, Section of Orthodontics, School of Dentistry, University of California, Los Angeles, CA 90095, USA; 3 Department of Surgery, David Geffen School of Medicine, University of California, Los Angeles, CA 90095, USA

**Keywords:** osteoarthritis, cartilage, whole transcriptome sequencing, biomarker, gender

## Abstract

As the leading cause of disability, osteoarthritis (OA) affects people of all ages, sexes, and races. With the increasing understanding of OA, the sex differences have attracted specific attention as the burden of OA is greater in women. There is no doubt that gender-specific OA management has great potential for precision treatment. On the other hand, from the marketing aspect, a medication targeting the OA-responsive biomarker(s) shared by both genders is more favorable for drug development. Thus, in the current study, a published transcriptome dataset of knee articular cartilage was used to compare OA and healthy samples for identifying the genes with the same significantly different expression trend in both males and females. With 128 genes upregulated and 143 genes downregulated in both OA males and females, 9 KEGG pathways have been enriched based on the current knowledge, including ‘renal cell carcinoma,’ ‘ECM-receptor interaction,’ ‘HIF-1 signaling pathway,’ ‘MicroRNAs in cancer,’ ‘focal adhesion,’ ‘Relaxin signaling pathway,’ ‘breast cancer,’ ‘PI3K-Akt signaling pathway,’ and ‘human papillomavirus infection.’ Here, we explore the potential impacts of these clusters in OA. We also analyze the identified ‘cell plasma membrane related genes’ in-depth to identify the potential chondrocyte cell surface target(s) of OA management.

## 1. Introduction

Osteoarthritis (OA) is the most common form of arthritis and a leading cause of disability [1], resulting in a tremendous burden on the affected individuals, healthcare systems, and socioeconomic costs. A recently published epidemiological report reveals that, globally, the age-standardized point prevalence and annual incidence rate of OA in 2017 were 3754.2 and 181.2 per 100,000, a boost of 9.3% and 8.2% from 1990, respectively [1]. With increased life expectancy and the inflated global population, OA-responsive financial disaster is foreseeable escalating worldwide problem [1].

Due to the fact that females have a higher prevalence of OA than males [1,2], ‘sex differences’ [2,3,4,5,6,7] and ‘gender-specific’ [8,9,10,11] started to attract more and more attention in the field of OA [12]. Women also experience more significant functional disability than men. Besides, compared with men, women are more likely to seek knee OA-related healthcare, such as narcotic or nonnarcotic analgesics, corticosteroid administration, hyaluronic acid injection, knee magnetic resonance imaging, physical therapy, and occupational therapy preceding total knee arthroplasty [13]. No doubt, fully understanding the sex-related differences of OA could significantly aid in comprehensively dissecting the underlying mechanism of the disease and eventually developing safe and efficient therapeutic approaches to OA treatment.

Despite the efforts made in the last several decades, there are currently no approved disease-modifying osteoarthritis drugs (DMOADs) that can prevent, stop, or even restrain OA progression [14,15,16]. The current treatments in clinical settings, such as the analgesics and corticosteroids mentioned above, are palliative and, unfortunately, end with the painful procedure of joint replacement. Thus, the Osteoarthritis Research Society International (OARSI) still recognizes OA as an incurable condition [15]. What is more, multiple adverse side-effects in the musculoskeletal, cardiovascular, and gastrointestinal systems [17,18,19] challenge the long-term usage of non-steroidal anti-inflammatory drugs (NSAIDs) and glucocorticoids as the safe treatment options for OA. Recently, the pro-chondrogenic growth factors turned out to be the hot points for seeking new OA therapeutics [20,21,22,23,24,25,26]. However, the prospect of strategies using well-known pro-chondrogenic growth factors as treatments for arthritis does not appear to be optimistic either. For example, a recent double-blind, phase 3 clinical trial shows that the application of transforming growth factor (TGF)β1-overexpressing chondrocytes does not have statistically significant effects for OA treatment in comparison with a placebo, even with the sacrifice of inducing multiple adverse events including peripheral edema (9%), arthralgia (8%), joint swelling (6%), and injection site pain (5%) [27]. Therefore, there is an emerging demand for novel DMOADs that simultaneously inhibit inflammation and promote cartilage regeneration. 

As mentioned previously, multiple studies have highlighted that OA pathogenesis, epidemiology, and disease characteristics are different between males and females; thus, when developing novel treatment targets of OA, we should also consider the gender-related factors. However, no matter if the studies were conducted in cell cultures in vitro or in animal models in vivo, most of the investigations intending to uncover OA mechanisms have not considered ‘Sex as a Biological Variable’ (SABV) to date [2]. For instance, from 2011 through 2019, only 32–40% of publications concerning knee OA evaluated sex-specific outcomes [12]. With the unignitable weight of sex, the current failure to identify an efficient OA treatment strategy might consciously exist until we take the sex into consideration. There is no doubt that gender-specific OA management has great potential for precision treatments. On another hand, from the marketing aspect, a medication targeting the OA-responsive biomarker(s) shared by both genders is more favorable for drug development. Therefore, publicly available transcriptome datasets of knee articular cartilage derived from healthy and OA donors were analyzed in the current study to gain insight into developing the new generation of DMOADs that could potentially benefit both genders.

## 2. Results

### 2.1. Initial Evaluation of the Cartilage RNA-seq Data Set

Using the keywords ‘osteoarthritis’ and ‘cartilage’ in the https://www.ncbi.nlm.nih.gov/gds database with the selection of ‘*Homo sapiens*’ under the column of ‘Top Organisms’ and ‘Expression profiling by high throughput sequencing’ under the column of ‘study type,’ 31 series were identified. After reviewing all these datasets to check if they provided the sex information of the donors, one series (GSE114007) containing transcriptome data of human knee cartilage from 18 healthy (5 females, 13 males) and 20 OA (11 females, 9 males) samples was included in the current study. The sample information is listed in Supplementary Table S1.

**Figure 1:**
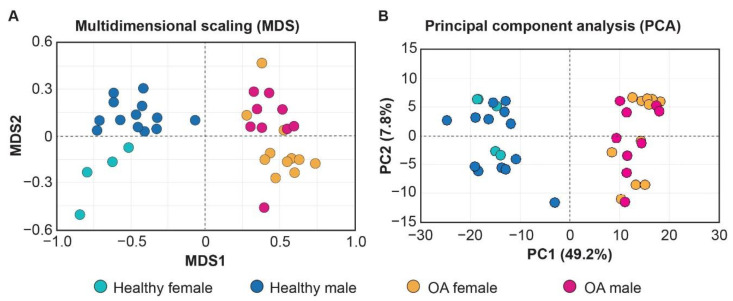
Multidimensional scaling (MDS) (**A**) and principal component analysis (PCA) (**B**) distinguish transcriptome of healthy and osteoarthritis (OA) cartilage samples included in the current study.

Both the multidimensional scaling (MDS) and principal component analysis (PCA) on the transcriptome profiling could distinguish the healthy and OA samples (Figure 1), while only MDS exhibits a trend to separate male and female samples (MDS2; Figure 1A), which highlights the difficulty for developing gender-specific OA-treatments.

### 2.2. Identification of the Common OA-Responsive Genes in Both Males and Females

**Figure 2:**
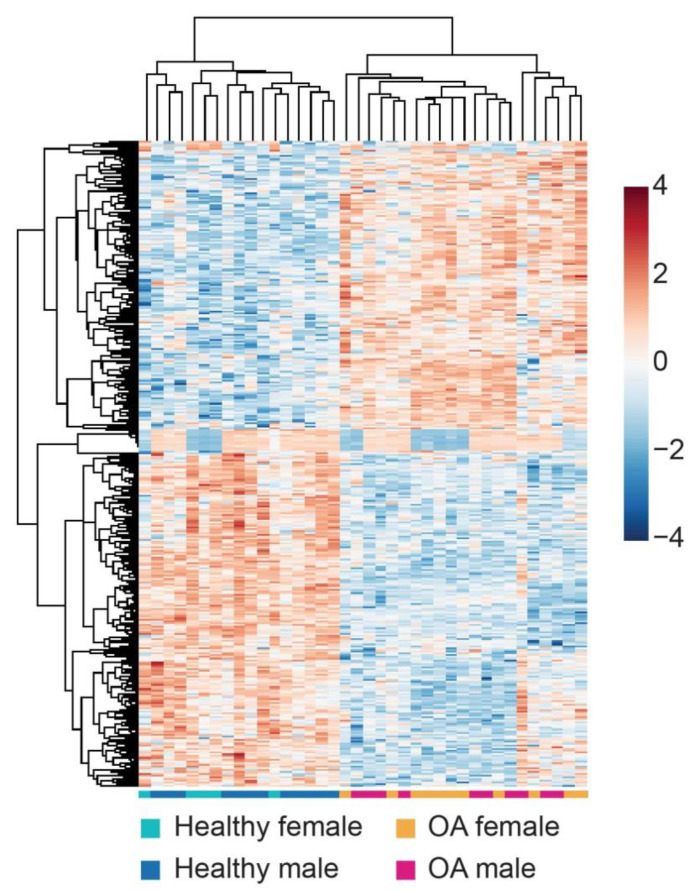
The heatmap visualizes the OA-responsive differential expressed genes (DEGs) in knee cartilage samples derived from both male and female donors.

**Figure 3:**
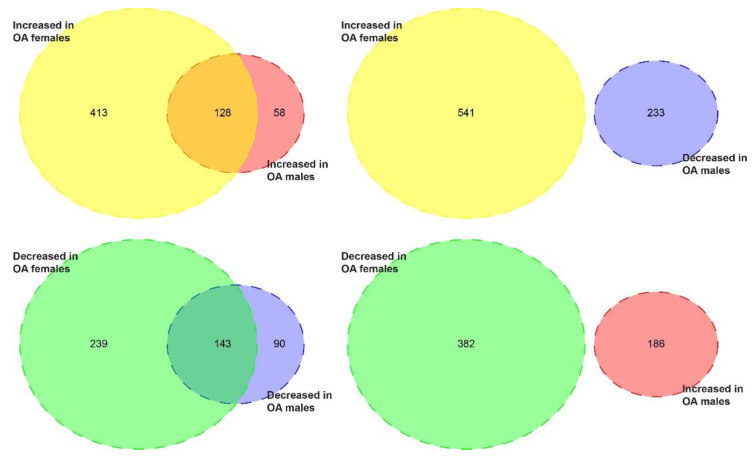
The Venn diagrams visualize the OA-responsive DEGs with the same or different expression trends in male and female cartilage samples. There are 541 increased and 382 decreased DEGs in female cartilage in response to OA, while there are 186 increased and 233 decreased DEGs in male cartilage in response to OA. When comparing DEGs in both genders, there are 128 upregulated DEGs and 143 downregulated DEGs shared by both genders. No gene exhibited different expression trends in males and females in response to OA.

The heatmap of the differential expression genes (DEGs) in the articular cartilage of the knee derived from male and female donors visualized the gene expression changes between healthy and OA samples (Figure 2). Overall, the healthy samples could be easily distinguished from OA samples, but there were no distinct male and female clusters. Comparing the gene alternation in the cartilage of each gender, females display more gene alteration in response to OA than males: 923 DEGs (541 increased, 382 decreased) were identified when comparing OA female samples to their healthy counterparts versus 419 OA-responsive DEGs (186 increased, 233 decreased) in male samples (Figure 3, Supplementary Tables S2 and S3). Moreover, we recognized 128 upregulated and 143 downregulated DEGs shared by both genders (Figure 3, Supplementary Tables S4 and S5). Besides, no gene exhibited different expression trends in males and females in response to OA (Figure 3).

### 2.3. Pathway Enrichment

**Figure 4:**
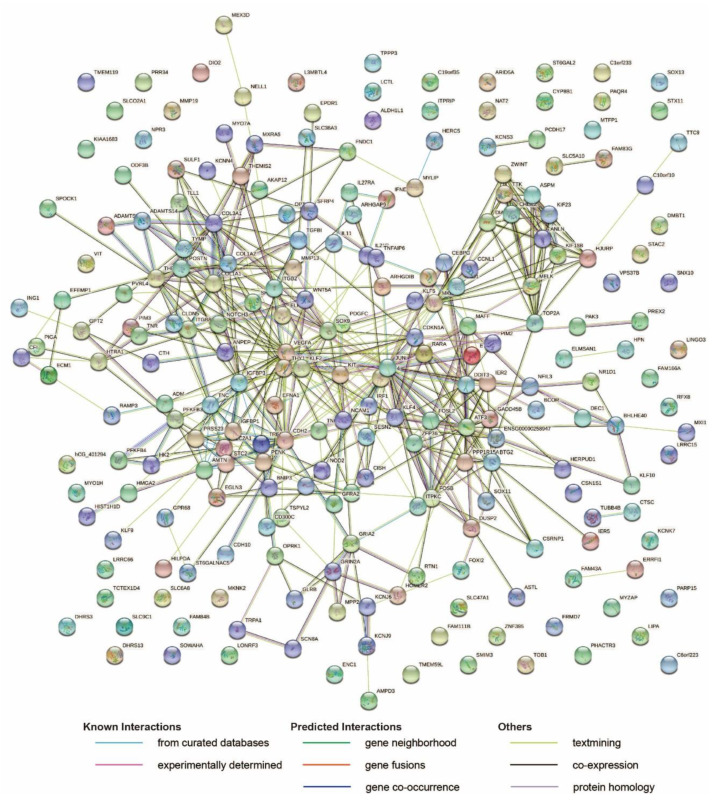
The protein–protein interaction network generated on the SPRING server against the *Homo sapiens* gene database. The STRING server identified 230 genes among the input of the 271 common DEGs in cartilage shared by both genders. The network nodes represent proteins. The empty nodes display the proteins without a known or predicted 3D structure, while the filled nodes illustrate those with a known or predicted 3D structure. The edges represent protein–protein associations: light blue–known interactions from curated databases; burgundy–experimentally determined known interactions; green–predicted interactions by gene neighborhood; orange–predicted interactions by gene fusions; dark blue–predicted interactions by gene co-occurrence; yellow–text mining; black–co-expression; purple–protein homology.

Firstly, pathway enrichment was conducted by the STRING networks [28] to gain insight into the potential underlying regulation pathways. Two hundred and thirty of the total 271 DEGs could be recognized by the STRING server (https://string-db.org/cgi/input?sessionId=bSVIOAB7JrVf&input_page_show_search=on; Figure 4), and 9 KEGG pathways were successfully enriched with a false discovery rate (FDR) smaller than 0.05 (Table 1). The ‘ECM-receptor interaction’ pathway was recognized as expected. In addition, ‘HIF-1 signaling pathway,’ ‘Focal adhesion,’ ‘Relaxin signaling pathway,’ and ‘PI3K-Akt signaling pathway’ were identified; these should be considered high priority targets for the further investigation as targets of OA shared by both genders. Surprisingly, ‘renal cell carcinoma,’ ‘MicroRNAs in cancer,’ ‘breast cancer,’ and ‘human papillomavirus infection’ were also enriched.

### 2.4. Cellular Component Enrichment

From a drug development stand point, the molecules located in the cell surface should be the first line targets, as they play critical roles in cell–cell interactions, nutrient and metabolite transportation, the translation of extracellular signals into intracellular response and vice versa, and, most importantly, due to their accessibility [29]. Thus, the enrichment of cellular components was also performed by STRING in the current study.

There were 21 genes grouped under ‘cell surface’ by the STRING server (Table 2). To further uncover these cell surface-related molecules’ potential function, the Uniprot database [30] was used to detail cellular locations and bioprocesses of these molecules (Table 3). Based on the Uniprot database, 13 among these 21 genes clustered by STRING, including Aminopeptidase N (ANPEP), Cadherin-2 (CDH2), Glutamine receptor 2 (GRIA2), Glutamate receptor ionotropic, NMDA 2A (GRIN2A), Serine protease hepsin (HPN), Integrin beta-2 (ITGB2), Integrin beta-8 (ITGB8), Mast/stem cell growth factor receptor Kit (KIT), Neural cell adhesion molecule 1 (NCAM1), Nucleotide-binding oligomerization domain-containing protein 2 (NOD2), Platelet-derived growth factor C (PDGFC), Receptor activity-modifying protein 3 (RAMP3), and Thy-1 membrane glycoprotein (THY1), encode a protein expressed on the plasma membrane.

It is worth noting that one of the plasma membrane expressed proteins, ITGB8, is associated with 4 of the 9 enriched KEGG pathways, including ‘ECM-receptor interaction,’ ‘focal adhesion,’ ‘PI3K-Akt signaling pathway,’ and ‘human papillomavirus infection’ (Table 1). This finding indicates that ITGB8 might be a key regulator for several cellular events and pathways in the cartilage during OA for both genders. Indeed, ITGB8 is already known to be associated with cartilage development (Table 3). Thus, ITGB8 might be considered one of the main targets for further OA treatment strategy investigations.

On the other hand, among the identified plasma membrane expressed proteins, ITGB8 is the only protein known to function in cartilage development or (re)generation based on the Uniprot database. As inflammation is a major event during OA, it is no surprise to recognize several proteins in the current study that participate in the inflammatory response, such as ANPEP (which has a function in neutrophil degranulation), ITGB2 (which has a function in leukocyte migration in the inflammatory response), KIT (which has a function in B cell differentiation and T cell differentiation), and NOD2 (which has a function in the cytokine production involved in the immune response; interleukin (IL)-1-mediated signaling pathway; macrophage apoptotic process; B cell activation; as well as IL10, IL17, IL1β, IL6, and IL8 production) (Table 3).

Unexpectedly, several proteins characterized as important neural event regulators were also displayed in the enrichment. For example, CDH2 regulates brain and cerebral cortex development, glial cell differentiation, and neuroligin clustering in postsynaptic membrane assembly; GRIA2 and GRIN2A participate in chemical synaptic transmission and regulation of NMDA receptor activity; and NCAM1 is important in commissural neuron axon guidance (Table 3). Since cartilage has no nerve intervention [31], the function of these neural related proteins on cartilage, especially during OA progression, warrants further investigation.

### 2.5. OA-Responsive Genes with Different Fold Change in Females and Males

**Figure 5:**
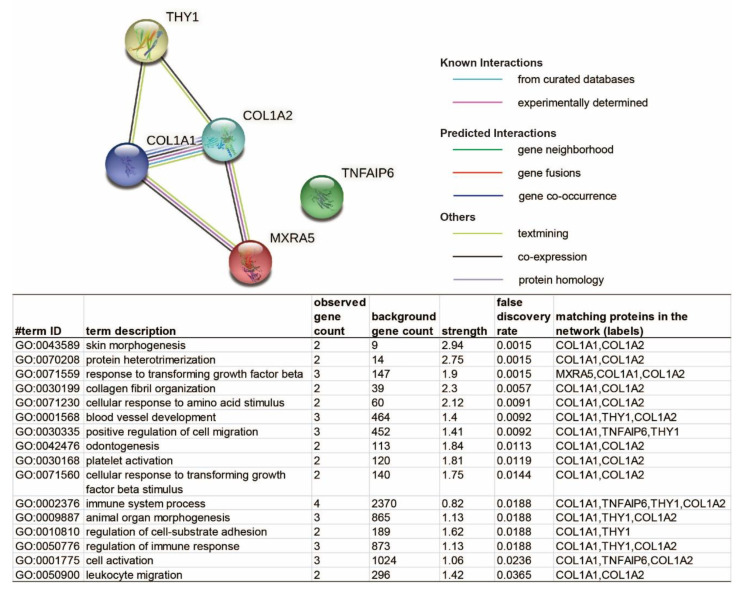
The STRING server displayed the protein–protein interaction network among the five common OA-responsive DEGs, which have significantly different multiples of change in male and female cartilage. The network nodes represent proteins, while filled nodes are for the proteins with known or predicted 3D structures. Edges represent protein–protein associations. The list contains identified pathways with a false discovery rate (FDR) of less than 0.05.

It is possible that, although some genes have a similar alteration trend in both genders, their expression fold change in response to OA may be significantly different in males and females. As listed in Table 4, we identified five genes that upregulated in both male and female OA cartilage tissues, including collagen type I alpha 1 chain (COL1A1), collagen type I alpha 2 chain (COL1A2), matrix remodeling associated 5 (MXRA5), THY1, TNF alpha induced protein 6 (TNFAIP6), which exhibited significantly higher OA-responsive increases in females than that found in males. For instance, both COL1A1 and COL1A2 are significantly higher in females than males, aligning with the observation that female patients tend to have more advanced cartilage degradation than male patients during OA [32]. Also, based on the functional information collected in the Uniprot database, both THY1 and TNFAIP6 participate in the inflammatory response (Supplementary Table S6), indicating that the female patients may have more intense inflammation in the cartilage than male patients during OA. The potential interaction of these five genes was also analyzed by the STRINGs server, and four of them were enriched in a biological process termed the ‘immune system process’ (Figure 5). This result further emphasizes the differently aroused levels of the immune response in males and females during OA, which potentially contributes to the phenomenon that female patients tend to have more severe OA symptoms than males [33]. Therefore, these genes and their encoded proteins may represent another set of targets for gender-specific OA-therapies.

## 3. Discussion

In the current study, several commonly used OA biomarkers have been detected in the cartilage of both genders, including the upregulated a disintegrin and metalloproteinase with thrombospondin-like motifs 7 (ADAMTS7) [34], ADAMTS14 [34], COL1A1, COL1A2, and matrix metallopeptidase 13 (MMP13) [35,36], as well as the downregulated sex-determining region Y box 9 (SOX9) [37]. In addition, four of eight hub genes recently identified by Zhang et al. [38], including Periostin (POSTN), Collagen Type III Alpha 1 Chain (COL3A1), COL1A1, and COL1A2, were identified in the current study. These results demonstrate the reliability of the current study.

It is worth noting that female cartilage demonstrated a significantly higher elevation of COL1A1 and COL1A2 in response to OA than male cartilage. Clinically, when patients seek treatment, females have more debilitating pain and experience more advanced OA stages than their male counterparts [32]. Our current finding proved that, at least for the donors included in the current study, women tend to have more fibrocartilage containing type I collagen than men during OA. Simultaneously, female OA cartilage displayed a more severe increase in TNFAIP6, THY1, and MXRA5. TNFAIP6 (also known as TSG-6), which has been demonstrated to be highly expressed in damaged articular and meniscal cartilage and cytokine-treated chondrocytes [39]. At the same time, THY1 (also known as CD90), a well-known inflammation regulator [40] and fibroblast-like cell surface marker [41], is also proved to be highly expressed in OA cartilage and could be induced by inflammatory cytokine interleukin-1β [42]. The reports about MXRA5 in OA cartilage are limited, but it has been identified in the synovial fluid of patients with OA [43]. Thus, the female OA cartilage has a more robust OA-responsive arousal of fibrocartilage-related and inflammation-related biomarkers than the male OA cartilage, which may partially explain why female patients tend to have more severe OA symptoms than males [33].

Besides, the enriched biological processes include ‘response to transforming growth factor beta’ based on proteins MXRA5, COL1A1, and COL1A2 (Figure 5). In fact, transforming growth factor-beta (TGF-β) family members have been intensively studied for OA therapeutic intervention [44,45]. However, the clinical trial results of TissueGene-C (TG-C), a cell and gene therapy for knee OA consisting of non-transformed and retrovirally transduced TGF-β1-overexpression chondrocytes (3:1), are not promising [27]. Considering the findings in the current study, we infer that the efficiently effective dose of TGF-β might be different in females and males.

Interestingly, we also observed several tumor-related clusters, including ‘renal cell carcinoma,’ ‘microRNAs in cancer,’ and ‘breast cancer’ from the enrichment of the common DEGs shared by female and male OA cartilage. Indeed, a recent cohort study reported that elderly patients with knee or hip OA have higher risks of melanoma, renal cell cancer, and cancer of the bladder, breast, uterus, and prostate [46], supporting our current discovery. This association between OA and tumorigenic risk may be attributed to the chronic usage of NSAIDs; however, further investigation is needed to uncover the full story.

The effects of hypoxia inducible factor-1 alpha (HIF-1α) and HIF-2α in OA have also been investigated for decades [47]. In brief, during OA, the hypoxic microenvironment could induce endoplasmic reticulum stress in chondrocytes and further disturb extracellular matrix (ECM) secretion. Although HIF-1α and HIF-2α mediate chondrocytes’ response to hypoxia [47,48], we did not detect HIF-1α or HIF-2α as the common DEGs shared by both genders in this study. Instead, we did detect several regulators or target genes of the HIF-1 signaling pathway, such as Egl-9 Family Hypoxia Inducible Factor 3 (EGLN3) [49], Hexokinase-2 (HK2) [50,51], Cyclin-dependent kinase inhibitor 1 (CDKN1A) [52], Solute carrier family 2, facilitated glucose transporter member 1 (SLC2A1) [53], 6-phosphofructo-2-kinase/fructose-2,6-bisphosphatase 3 (PFKFB3) [53], and Vascular Endothelial Growth Factor A (VEGFA) [50,52]. Thus, the regulation of hypoxia [54] and endoplasmic reticulum stress [55] could be one of the potential targets of OA management in both genders.

In addition, ‘Relaxin signaling pathway’ is one of the enriched clusters. Through binding to its receptor in various tissues and via medication by signaling pathways, Relaxin has regulatory effects on the musculoskeletal and other systems [56]. The Relaxin signaling pathway has many biological actions, including anti-fibrotic, vasodilatory, angiogenic, anti-inflammatory, anti-apoptotic, and organ protective effects across various tissues [57]. Specific to cartilage, Relaxin appears to decrease knee articular cartilage stiffness [56]. However, knowledge about the Relaxin signaling pathway in OA is still largely lacking.

The PI3K-Akt signaling pathway was also recognized by STRING. The key molecules involved in this signaling pathway are receptor tyrosine kinase (RTKs), phosphatidylinositol 3-kinase (PI3K), phosphatidylinositol-4,5-bisphosphate (PIP2), phosphatidylinositol-3,4,5-bisphosphate (PIP3), and AKT/protein kinase B. As an intracellular signal transduction pathway that promotes metabolism, proliferation, cell survival, growth, and angiogenesis in response to extracellular signals, the PI3K/Akt signaling pathway participates in stem cell chondrogenesis [58], chondrocyte protection [58], chondrocyte apoptosis [59,60], chondrocyte hypertrophic and fibrotic differentiation [61], cartilage degradation [62], and inflammation regulation [63,64]. Besides, the effects of the PI3K/Art signaling pathway are not limited to cartilage. For instance, it also regulates subchondral bone dysfunction and synovial inflammation [65]. Thus, the PI3K-Art signaling pathway could be a strong candidate for further OA related investigations.

As mentioned above, the surface molecules could be the primary targets for drug development because of their critical roles and, more crucially, their accessibility [29]. ITGB8 is a cell plasma membrane protein that matched with four of the nine enrichment pathways, including ‘ECM-receptor interaction,’ ‘focal adhesion,’ ‘PI3K-Art signaling pathway,’ and ‘human papillomavirus infection.’ The integrin family members play a major role in mediating the interaction between cells and the extracellular matrix (ECM) [66]. Using multiple chondrogenic models, a previous in vitro investigation demonstrated that ITGB8 was consistently upregulated in human mesenchymal stem cells (MSCs) during chondrogenic differentiation [67]. On the other hand, knocking down ITGB8 markedly impacted MSCs’ chondrogenic differentiation [67]. A recent publication also indicates that ITGB8 plays a key role in chondrocyte sheet attachment and the in ECM synthesis in cartilage [68]. In combination, ITGB8 seems to have a beneficial effect on cartilage. Interestingly, we noticed the upregulation of ITGB8 in both male and female OA cartilage, suggesting upregulation of ITGB8 may have a protect/repair function on the cartilage in response to OA progression. However, the detailed functions of ITGB8 and whether it can be the master target that regulates several important signaling pathways during OA treatment are still open questions that remain to be answered.

Finally, several neural cell membrane proteins’ levels were significantly altered in response to OA in both male and female cartilage. Since cartilage has no nerve intervention [31], the OA-responsive adaption of gene expression is more than likely from chondrocytes. Meanwhile, accumulating studies demonstrate that the crosstalk between skeletal and neural tissue is critical for skeletal health [68,69]. For example, there is bidirectional communication between the sensory neurons and osteoblasts through glutamate, substance P, and ATP [70]. Unfortunately, since the skeletal–neuron crosstalk evaluation is still in its infancy, most investigations have focused on bone and muscle as these two tissues have nerve innervation [71,72]. So far, based on the authors’ thorough literature search with the keywords ‘cartilage or chondrocyte + nerve or neural + crosstalk,’ there is no publicly available literature dissecting the interaction between articular cartilage and the neural system. The only available report we could access revealed the elevation of Chemotactic cytokine ligand 2 (CCL2) and nerve growth factor (NGF) in the joint during OA mediate inflammation and pain [73]. Based on the current findings that cartilage expresses neural-related cell membrane proteins and that the expression of these proteins amended in response to OA, whether cartilage–nerve crosstalk exists or the neural-related proteins have some unrevealed functions in cartilage generation and maintenance are definitely interesting topics to be investigated in-depth. 

Although the main objective of this investigation is to analyze OA-related gene expression changes in cartilage shared by both genders, we are aware that both age and body mass index (BMI) are also important confounders. Unfortunately, although healthy and OA donors’ body mass is not statistically different, individual BMI information was not provided in the original study [74]. We also noticed that both male and female OA donors were older than their healthy counterparts (Supplementary Table S1), which agrees with the clinical observation that OA’s incidence increases along with age [75]. On the other hand, this age difference may lead to the false-positive identification of DEG to some degree, as discussed in the original investigation [74]. Considering that the OA samples were collected in the clinic for a single study, the sample size is insufficient for us to conduct multivariate analysis involving the donor’s age and BMI for result adjustment. It is potentially a major limitation of the original investigation, as well as our current study, and highlights the importance of worldwide collaboration on sample collection and complete data sharing.

## 4. Materials and Methods 

The sequence read archive (SRA) data of healthy and OA human knee cartilage (GEO accession number: GSE114007) RNA-seq data were downloaded from https://www.ncbi.nlm.nih.gov/sra. Specifically, normal human knee cartilage tissues were procured by tissue banks from donors without a history of joint disease or trauma and processed within 24–48 hours post mortem. OA-affected cartilage was harvested from the tissue removed during knee replacement surgery. Cartilage was stored at −20 °C in Allprotect Tissue Reagent (Qiagen, Valencia, CA, USA) immediately after harvest until RNA extraction [74]. Data analyses were performed on the Galaxy platform (UseGalaxy.org, [76]). The FASTQC RNA-seq reads were aligned to the human genome (GRCh38) using the HISAT2 aligner (Galaxy Version 2.1.0+galaxy 5) with default parameters [77]. Raw counts of sequencing read for the feature of genes were extracted by featureCounts (Galaxy Version 1.6.4+galaxy1) [78]. Then, the limma package (Galaxy version 3.38.3+galaxy3) was used to identify DEGs with its voom method [79,80]. The expressed genes were selected as their counts per million (CPM) value were not less than one in at least two samples across the entire experiment, while lowly expressed genes were removed for the flowing analyses. Quasi-likelihood F-tests (ANOVA-like analysis) were achieved to identify DEGs [81]. Genes with a fold change (FC) of more than 2 and FDR less than 0.01 were assigned as DEGs. Heatmap, MDS, PCA, and Venn diagram construction were conducted in R (version 3.6.3, Free Software Foundation, Boston, MA, USA) [82] with the packages pheatmap (version 1.0.12), vegan (version 2.56), ggplot2 (version 3.3.0) and VennDiagram (version 1.6.20). The pathway enrichment of identified DEGs was performed against the STRING network [28]. In addition, the summary of the known biofunctions for these genes was searched in the Uniprot database [30] for manually functional annotation.

## 5. Conclusions

In summary, our current study identified several novel genes and signaling pathways as the potential targets for further OA investigations, which will benefit both genders. In addition, several neural-related cell surface proteins whose expression is altered in response to OA in both genders’ cartilage were also recognized, indicating the potential neuro–cartilage interaction. Last but not least, genes which have a significantly different fold change in male and female cartilages during OA were also evaluated, providing a potential explanation for different OA symptom severity and treatment responses in male and female patients. Overall, we hope our work could help direct the further development of DMOADs, which could truly benefit both male and female OA patients.

## Figures and Tables

**Table 1:** KEGG pathway enrichment results.

#Term ID	Term Description	Observed Gene Count	Background Gene Count	Strength	False Discovery Rate	Matching Proteins (Labels)
hsa05211	Renal cell carcinoma	6	68	0.88	0.0144	EGLN3, PAK3, JUN, CDKN1A, SLC2A1, VEGFA
hsa04512	ECM-receptor interaction	6	81	0.8	0.0208	ITGB8, COL1A1, TNC, COL1A2, THBS2, TNR
hsa04066	HIF-1 signaling pathway	7	98	0.78	0.0144	EGLN3, MKNK2, HK2, VEGFA, CDKN1A, SLC2A1, PFKFB3
hsa05206	MicroRNAs in cancer	10	149	0.76	0.0034	KIF23, NOTCH3, TNC, DDIT4, SHC4, TNR, IRS2, CDKN1A, HMGA2, VEGFA
hsa04510	Focal adhesion	11	197	0.68	0.0034	ITGB8, COL1A1, TNC, COL1A2, SHC4, PAK3, THBS2, TNR, JUN, PDGFC, VEGFA
hsa04926	Relaxin signaling pathway	7	130	0.66	0.0353	COL1A1, MMP13, COL1A2, COL3A1, SHC4, JUN, VEGFA
hsa05224	Breast cancer	7	147	0.61	0.0476	GADD45B, NOTCH3, WNT5A, KIT, SHC4, JUN, CDKN1A
hsa04151	PI3K-Akt signaling pathway	12	348	0.47	0.0353	ITGB8, COL1A1, TNC, COL1A2, KIT, DDIT4, THBS2, EFNA1, CDKN1A, PDGFC, VEGFA, TNR
hsa05165	Human papillomavirus infection	11	317	0.47	0.0404	ITGB8, COL1A1, IRF1, NOTCH3, WNT5A, TNC, COL1A2, THBS2, TNR, CDKN1A, VEGFA

**Table 2:** Cellular Component enrichment results.

#Term ID	Term Description	Observed Gene Count	Background Gene Count	Strength	False Discovery Rate	Matching Proteins (Labels)
GO:0005584	collagen type I trimer	2	2	1.93	0.0365	COL1A1, COL1A2
GO:1990622	CHOP-ATF3 complex	2	2	1.93	0.0365	ATF3, DDIT3
GO:0005583	fibrillar collagen trimer	3	11	1.37	0.0322	COL1A1, COL1A2, COL3A1
GO:0062023	collagen-containing extracellular matrix	10	144	0.77	0.0014	COL1A1, TNC, COL1A2, COL3A1, AMTN, THBS2, TNR, VIT, EFEMP1, TGFBI
GO:0031012	extracellular matrix	17	283	0.71	3.67 × 10^−5^	COL1A1, MMP13, WNT5A, TNC, COL1A2, COL3A1, MMP19, AMTN, THBS2, OPTC, TNR, ECM1, VIT, POSTN, EFEMP1, TGFBI, VEGFA
GO:0005788	endoplasmic reticulum lumen	15	299	0.63	0.00074	COL1A1, CTSC, WNT5A, STC2, TNC, CDH2, IGFBP1, PRSS23, COL1A2, COL3A1, PENK, AMTN, IGFBP3, ADAMTS7, PDGFC
GO:0009986	cell surface	21	690	0.41	0.0074	ITGB8, RAMP3, RARA, HILPDA, SULF1, HPN, WNT5A, CDH2, THY1, KIT, GRIA2, ANPEP, NOD2, TNR, ADAMTS7, GRIN2A, ITGB2, SFRP4, PDGFC, VEGFA, NCAM1
GO:0005615	extracellular space	27	1134	0.31	0.0322	COL1A1, TNFAIP6, HILPDA, SULF1, MMP13, IL11, WNT5A, IGFBP1, KIT, COL1A2, ANPEP, COL3A1, HBB, HTRA1, TNFSF15, POSTN, IGFBP3, EFEMP1, CFI, SPOCK1, SFRP4, TGFBI, IFNE, PDGFC, ADM, LINGO3, VEGFA
GO:0005576	extracellular region	55	2505	0.27	0.00074	TLL1, EPDR1, COL1A1, CTSC, TNFAIP6, TREM1, CSN1S1, HILPDA, SULF1, MMP13, KIF23, NOTCH3, IL11, WNT5A, TNC, IGFBP1, THY1, KIT, FNDC1, COL1A2, NELL1, ANPEP, COL3A1, MMP19, PENK, HBB, AMTN, TUBB4B, THBS2, OPTC, TNR, EFNA1, DMBT1, HTRA1, ECM1, ADAMTS14, TNFSF15, DHRS13, VIT, POSTN, IGFBP3, ADAMTS7, ARHGAP9, EFEMP1, CFI, SPOCK1, AMPD3, SFRP4, TGFBI, IFNE, PDGFC, ADM, LINGO3, VEGFA, NCAM1

**Table 3:** Twenty-one common DEGs related to ‘cell surface’.

Symbol	Gene Name	Expression	Function in Uniprot
ANPEP	Aminopeptidase N	plasma membrane	angiogenesis; cell differentiation; neutrophil degranulation; peptide catabolic process; proteolysis
CDH2	Cadherin-2	plasma membrane	adherents junction organization; blood vessel morphogenesis; brain development; brain morphogenesis; calcium-dependent cell–cell adhesion via plasma membrane cell adhesion molecules; cell adhesion; cell–cell adhesion; cell–cell adhesion mediated by cadherin; cell–cell adhesion via plasma-membrane adhesion molecules; cell migration; cellular protein metabolic process; cerebral cortex development; glial cell differentiation; heterophilic cell–cell adhesion via plasma membrane cell adhesion molecules; homeostasis of number of cells; homophilic cell adhesion via plasma membrane adhesion molecules; negative regulation of canonical Wnt signaling pathway; neuroepithelial cell differentiation; neuroligin clustering involved in postsynaptic membrane assembly; neural stem cell population maintenance; positive regulation of MAPK cascade; positive regulation of muscle cell differentiation; positive regulation of synaptic vesicle clustering; post-translational protein modification; protein localization to plasma membrane; radial glial cell differentiation; regulation of axonogenesis; regulation of oligodendrocyte progenitor proliferation; regulation of postsynaptic density protein 95 clustering; regulation of synaptic transmission, glutamatergic; striated muscle cell differentiation; synapse assembly
GRIA2	Glutamate receptor 2	Endoplasmic reticulum; plasma membrane	chemical synaptic transmission; ionotropic glutamate receptor signaling pathway; regulation of NMDA receptor activity; signal transduction; synaptic transmission, glutamatergic
GRIN2A	Glutamate receptor ionotropic, NMDA 2A	plasma membrane	activation of cysteine-type endopeptidase activity; brain development; calcium ion transmembrane import into cytosol; chemical synaptic transmission; directional locomotion; dopamine metabolic process; excitatory chemical synaptic transmission; excitatory postsynaptic potential; glutamate receptor signaling pathway; learning or memory; long-term synaptic potentiation; memory; negative regulation of protein catabolic process; neurogenesis; positive regulation of apoptotic process; protein localization to postsynaptic membrane; regulation of NMDA receptor activity; regulation of synaptic plasticity; response to amphetamine; response to drug; response to ethanol; response to wounding; sensory perception of pain; serotonin metabolic process; sleep; startle response; visual learning
HPN	Serine protease hepsin	plasma membrane	basement membrane disassembly; cochlea morphogenesis; detection of mechanical stimulus involved in sensory perception of sound; hepatocyte growth factor receptor signaling pathway; negative regulation of apoptotic process; negative regulation of epithelial cell proliferation; negative regulation of epithelial to mesenchymal transition; pilomotor reflex; positive regulation by host of viral transcription; positive regulation of cell growth; positive regulation of gene expression; positive regulation of hepatocyte proliferation; positive regulation of plasminogen activation; positive regulation of thyroid hormone generation; potassium ion transmembrane transport; proteolysis; regulation of cell shape; response to thyroid hormone
ITGB2	Integrin beta-2	plasma membrane	aging; amyloid-beta clearance; apoptotic process; cell adhesion; cell adhesion mediated by integrin; cell–cell adhesion; cell–cell adhesion via plasma-membrane adhesion molecules; cell–cell signaling; cell-matrix adhesion; cell migration; cellular response to low-density lipoprotein particle stimulus; cytokine-mediated signaling pathway; endodermal cell differentiation; endothelial cell migration; extracellular matrix organization; heterotypic cell–cell adhesion; inflammatory response; integrin-mediated signaling pathay; leukocyte cell–cell adhesion; leukocyte migration; leukocyte migration involved in inflammatory response; microglial cell activation; natural killer cell activation; negative regulation of dopamine metabolic process; neutrophil chemotaxis; neutrophil degranulation; neutrophil migration; phagocytosis, engulfment; positive regulation of angiogenesis; positive regulation of leukocyte adhesion to vascular endothelial cell; positive regulation of neuron death; positive regulation of neutrophil degranulation; positive regulation of NF-kappaB transcription factor activity; positive regulation of nitric oxide biosynthetic process; positive regulation of prostaglandin-E synthase activity; positive regulation of protein targeting to membrane; positive regulation of superoxide anion generation; receptor clustering; receptor internalization; receptor-mediated endocytosis; regulation of cell shape; regulation of immune response; regulation of peptidyl-tyrosine phosphorylation; toll-like receptor 4 signaling pathway
ITGB8	Integrin beta-8	plasma membrane	cartilage development; cell adhesion; cell adhesion mediated by integrin; cell migration; extracellular matrix organization; ganglioside metabolic process; integrin-mediated signaling pathway; negative regulation of gene expression; placenta blood vessel development; positive regulation of angiogenesis; positive regulation of gene expression; regulation of transforming growth factor eta activation; vasculogenesis
KIT	Mast/stem cell growth factor receptor Kit	plasma membrane	actin cytoskeleton reorganization; activation of MAPK activity; B cell differentiation; cell chemotaxis; cellular response to thyroid hormone stimulus; cytokine-mediated signaling pathway; dendritic cell cytokine production; detection of mechanical stimulus involved in sensory perception of sound; digestive tract development; ectopic germ cell programmed cell death; embryonic hemopoiesis; epithelial cell proliferation; erythrocyte differentiation; erythropoietin-mediated signaling pathway; Fc receptor signaling pathway; germ cell migration; glycosphingolipid metabolic process; hematopoietic stem cell migration; hemopoiesis; immature B cell differentiation; inflammatory response; Kit signaling pathway; lamellipodium assembly; lymphoid progenitor cell differentiation; male gonad development; MAPK cascade; mast cell chemotaxis; mast cell cytokine production; mast cell degranulation; mast cell proliferation; megakaryocyte development; melanocyte adhesion; melanocyte differentiation; melanocyte migration; multicellular organism development; myeloid progenitor cell differentiation; negative regulation of programmed cell death; ovarian follicle development; peptidyl-tyrosine phosphorylation; pigmentation; positive regulation of cell migration; positive regulation of cell population proliferation; positive regulation of colon smooth muscle contraction; positive regulation of DNA-binding transcription factor activity; positive regulation of gene expression; positive regulation of kinase activity; positive regulation of long-term neuronal synaptic plasticity; positive regulation of MAPK cascade; positive regulation of MAP kinase activity; positive regulation of Notch signaling pathway; positive regulation of phosphatidylinositol 3-kinase activity; positive regulation of phosphatidylinositol 3-kinase signaling; positive regulation of phospholipase C activity; positive regulation of protein kinase B signaling; positive regulation of pseudopodium assembly; positive regulation of pyloric antrum smooth muscle contraction; positive regulation of receptor signaling pathway via JAK-STAT; positive regulation of small intestine smooth muscle contraction; positive regulation of tyrosine phosphorylation of STAT protein; positive regulation of vascular associated smooth muscle cell differentiation; protein autophosphorylation; regulation of bile acid metabolic process; regulation of cell population proliferation; regulation of cell shape; regulation of transcription by RNA polymerase II; response to cadmium ion; signal transduction; somatic stem cell division; somatic stem cell population maintenance; spermatid development; spermatogenesis; stem cell differentiation; stem cell population maintenance; T cell differentiation; tongue development; transmembrane receptor protein tyrosine kinase signaling pathway; visual learning
NCAM1	Neural cell adhesion molecule 1	plasma membrane; extracellular region or secreted	axon guidance; cell adhesion; commissural neuron axon guidance; interferon-gamma-mediated signaling pathway; MAPK cascade; regulation of semaphorin–plexin signaling pathway
NOD2	Nucleotide-binding oligomerization domain-containing protein 2	plasma membrane	activation of MAPK activity; cellular response to lipopolysaccharide; cellular response to muramyl dipeptide; cellular response to organic cyclic compound; cellular response to peptidoglycan; cytokine production involved in immune response; cytokine secretion involved in immune response; defense response; defense response to bacterium; detection of bacterium; detection of biotic stimulus; detection of muramyl dipeptide; innate immune response; interleukin-1-mediated signaling pathway; intracellular signal transduction; JNK cascade; maintenance of gastrointestinal epithelium; negative regulation of macrophage apoptotic process; nucleotide-binding oligomerization domain containing 2 signaling pathway; nucleotide-binding oligomerization domain containing signaling pathway; pattern recognition receptor signaling pathway; positive regulation of B cell activation; positive regulation of cytokine production involved in inflammatory response; positive regulation of dendritic cell antigen processing and presentation; positive regulation of dendritic cell cytokine production; positive regulation of epithelial cell proliferation; positive regulation of ERK1 and ERK2 cascade; positive regulation of gamma-delta T cell activation; positive regulation of I-kappaB kinase/NF-kappaB signaling; positive regulation of interleukin-10 production; positive regulation of interleukin-17 production; positive regulation of interleukin-1 beta production; positive regulation of interleukin-6 production; positive regulation of interleukin-8 production; positive regulation of JNK cascade; positive regulation of MAP kinase activity; positive regulation of NF-KappaB signaling; positive regulation of nitric-oxide synthase biosynthetic process; positive regulation of Notch signaling pathway; positive regulation of oxidoreductase activity; positive regulation of phosphatidylinositol 3-kinase activity; positive regulation of prostaglandin-endoperoxide synthase activity; positive regulation of prostaglandin-E synthase activity; positive regulation of protein k63-linked ubiquitination; positive regulation of stress-activated MAPK cascade; positive regulation of transcription by RNA polymerase II; positive regulation of tumor necrosis factor production; positive regulation of type 2 immune response; regulation of inflammatory response; response to muramyl dipeptide; response to nutrient
PDGFC	Platelet-derived growth factor C	Extracellular region or secreted; Nucleus; Cytosol; Plasma membrane	activation of transmembrane receptor protein tyrosine kinase activity; animal organ morphogenesis; bone development; cellular response to amino acid stimulus; central nervous system development; digestive tract development; embryonic organ development; platelet-derived growth factor receptor signaling pathway; positive regulation of cell division; positive regulation of cell migration; positive regulation of cell population proliferation; positive regulation of cold-induced thermogenesis; positive regulation of ERK1 and ERK2 cascade; positive regulation of fibroblast proliferation; positive regulation of MAP kinase activity; positive regulation of phosphatidylinositol 3-kinase signaling; positive regulation of protein autophosphorylation; regulation of peptidyl-tyrosine phosphorylation
RAMP3	Receptor activity-modifying protein 3	plasma membrane	adenylate cyclase-activating G protein-coupled receptor signaling pathway; adrenomedullin receptor signaling pathway; amylin receptor signaling pathway; angiogenesis; calcium ion transport; cellular response to estradiol stimulus; cellular response to hormone stimulus; cross-receptor inhibition within G protein-coupled receptor heterodimer; G protein-coupled receptor signaling pathway; G protein-coupled receptor signaling pathway involved in heart process; intracellular protein transport; positive regulation of calcium ion import across plasma membrane; positive regulation of cell death; positive regulation of ERK1 and ERK2 cascade; positive regulation of gene expression; positive regulation of peptidyl-serine phosphorylation; positive regulation of protein kinase A signaling; positive regulation of protein kinase B signaling; positive regulation of protein localization to plasma membrane; positive regulation of receptor recycling; protein localization to plasma membrane; protein transport; receptor internalization; response to amyloid beta
THY1	Thy-1 membrane glycoprotein	plasma membrane	angiogenesis; cell–cell adhesion; cell–cell signaling; cytoskeleton organization; focal adhesion assembly; integrin-mediated signaling pathway; negative regulation of axonogenesis; negative regulation of cell migration; negative regulation of neuron projection regeneration; negative regulation of protein kinase activity; negative regulation of protein tyrosine kinase activity; negative regulation of T cell receptor signaling pathway; positive regulation of cellular extravasation; positive regulation of focal adhesion assembly; positive regulation of GTPase activity; positive regulation of heterotypic cell–cell adhesion; positive regulation of release of sequestered calcium ion into cytosol; positive regulation of T cell activation; protein autophosphorylation; receptor clustering; regulation of cell-matrix adhesion; regulation of Rho-dependent protein serine/threonine kinase activity; retinal cone cell development; T cell receptor signaling pathway
ADAMTS7	A disintegrin and metalloproteinase with thrombospondin motifs 7	Extracellular region or secreted	cellular response to BMP stimulus; cellular response to interleukin-1; cellular response to tumor necrosis factor; extracellular matrix organization; negative regulation of chondrocyte differentiation; proteolysis involved in cellular protein catabolic process
HILPDA	Hypoxia-inducible lipid droplet-associated protein	Extracellular region or secreted	autocrine signaling; cellular response to hypoxia; lipid droplet organization; positive regulation of cell population proliferation; positive regulation of cytokine production; positive regulation of lipid storage
SFRP4	Secreted frizzled-related protein 4	extracellular region or secreted	bone morphogenesis; canonical Wnt signaling pathway; cell differentiation; negative regulation of canonical Wnt signaling pathway; negative regulation of cell population proliferation; negative regulation of DNA-binding transcription factor activity; negative regulation of non-canonical Wnt signaling pathway; negative regulation of sodium-dependent phosphate transport; negative regulation of Wnt signaling pathway; phosphate ion homeostasis; positive regulation of apoptotic process; positive regulation of canonical Wnt signaling pathway; positive regulation of epidermal cell differentiation; positive regulation of gene expression; positive regulation of keratinocyte apoptotic process; positive regulation of receptor internalization; regulation of BMP signaling pathway; response to hormone
TNR	Tenascin-R	extracellular region or secreted	associative learning; axon guidance; cell adhesion; extracellular matrix organization; locomotory exploration behavior; long-term synaptic potentiation; negative regulation of cell–cell adhesion; negative regulation of synaptic transmission; neuromuscular process controlling balance; neuron cell–cell adhesion; positive regulation of synaptic transmission, glutamatergic; positive regulation of transmission of nerve impulse; synapse organization; telencephalon cell migration
VEGFA	Vascular endothelial growth factor A	extracellular region or secreted	activation of protein kinase activity; angiogenesis; artery morphogenesis; basophil chemotaxis; branching involved in blood vessel morphogenesis; branching morphogenesis of an epithelial tube; camera-type eye morphogenesis; cardiac muscle fiber development; cardiac vascular smooth muscle cell development; cell maturation; cell migration involved in sprouting angiogenesis; cellular response to hypoxia; cellular response to vascular endothelial growth factor stimulus; cellular stress response to acid chemical; commissural neuron axon guidance; coronary artery morphogenesis; coronary vein morphogenesis; cytokine-mediated signaling pathway; dopaminergic neuron differentiation; endothelial cell chemotaxis; epithelial cell differentiation; eye photoreceptor cell development; heart morphogenesis; induction of positive chemotaxis; in utero embryonic development; kidney development; lactation; lung development; lymph vessel morphogenesis; macrophage differentiation; mammary gland alveolus development; mesoderm development; monocyte differentiation; motor neuron migration; negative regulation of adherents junction organization; negative regulation of apoptotic process; negative regulation of blood–brain barrier permeability; negative regulation of cell–cell adhesion mediated by cadherin; negative regulation of cysteine-type endopeptidase activity involved in apoptotic process; negative regulation of establishment of endothelial barrier; negative regulation of gene expression; negative regulation of transcription by RNA polymerase II; nervous system development; outflow tract morphogenesis; ovarian follicle development; platelet degranulation; positive chemotaxis; positive regulation of angiogenesis; positive regulation of axon extension involved in axon guidance; positive regulation of blood vessel endothelial cell migration; positive regulation of blood vessel endothelial cell proliferation involved in sprouting angiogenesis; positive regulation of branching involved in ureteric bud morphogenesis; positive regulation of cell adhesion; positive regulation of cell division; positive regulation of cell migration; positive regulation of cell migration involved in sprouting angiogenesis; positive regulation of cell population proliferation; positive regulation of cell proliferation by VEGF-activated platelet derived growth factor receptor signaling pathway; positive regulation of cellular component movement; positive regulation of cold-induced thermogenesis; positive regulation of CREB transcription factor activity; positive regulation of DNA biosynthetic process; positive regulation of endothelial cell chemotaxis by VEGF-activated vascular endothelial growth factor receptor signaling pathway; positive regulation of endothelial cell migration; positive regulation of endothelial cell proliferation; positive regulation of ERK1 and ERK2 cascade; positive regulation of focal adhesion assembly; positive regulation of gene expression; positive regulation of histone deacetylase activity; positive regulation of leukocyte migration; positive regulation of MAP kinase activity; positive regulation of mast cell chemotaxis; positive regulation of mesenchymal cell proliferation; positive regulation of neuroblast proliferation; positive regulation of p38MAPK cascade; positive regulation of peptidyl-serine phosphorylation; positive regulation of peptidyl-tyrosine autophosphorylation; positive regulation of protein-containing complex assembly; positive regulation of protein kinase C signaling; positive regulation of protein kinase D signaling; positive regulation of protein localization to early endosome; positive regulation of protein phosphorylation; positive regulation of receptor internalization; positive regulation retinal ganglion cell axon guidance; positive regulation of sprouting angiogenesis; positive regulation of transcription by RAN polymerase II; positive regulation of transcription from RNA polymerase II promoter in response to hypoxia; positive regulation of trophoblast cell migration; positive regulation of vascular permeability; post-embryonic camera-type eye development; primitive erythrocyte differentiation; regulation of cell shape; regulation of nitric oxide mediated signal transduction; regulation of retinal ganglion cell axon guidance; regulation of transcription by RNA polymerase II; regulation of transcription by RNA polymerase II promoter in response to hypoxia; response to hypoxia; sprouting angiogenesis; surfactant homeostasis; tube formation; vascular endothelial growth factor receptor-2 signaling pathway; vascular endothelial growth factor receptor signaling pathway; vascular endothelial growth factor signaling pathway; vascular wound healing; vasculogenesis; VEGF-activated neuropilin signaling pathway
WNT5A	Protein Wnt-5a	Extracellular region or secreted	activation of GTPase activity; activation of JUN kinase activity; activation of MAPK activity; activation of protein kinase B activity; anterior/posterior axis specification, embryo; atrial septum development; axon guidance; canonical Wnt signaling pathway; cartilage development; cell fate commitment; cellular protein localization; cellular response to calcium ion; cellular response to interferon-gamma; cellular response to lipopolysaccharide; cellular response to retinoic acid; cellular response to transforming growth factor beta stimulus; cervix development; chemoattraction of serotonergic neuron axon; chemorepulsion of dopaminergic neuron axon; cochlea morphogenesis; convergent extension involved in axis elongation; convergent extension involved in organogenesis; embryonic skeletal system development; epithelial cell proliferation involved in mammary gland duct elongation; epithelial to mesenchymal transition; establishment of epithelial cell apical/basal polarity; establishment of planar polarity; excitatory synapse assembly; face development; genitalia development; heart looping; hematopoietic stem cell proliferation; hindgut morphogenesis; hypophysis morphogenesis; inhibitory synapse assembly; keratinocyte differentiation; kidney development; lateral sprouting involved in mammary gland duct morphogenesis; lens development in camera-type eye; lung development; male gonad development; mammary gland branching involved in thelarche; melanocyte proliferation; membrane organization; mesenchymal-epithelial cell signaling; mesodermal to mesenchymal transition involved in gastrulation; midgut development; negative regulation of apoptotic process; negative regulation of axon extension involved in axon guidance; negative regulation of BMP signaling pathway; negative regulation of canonical Wnt signaling pathway; negative regulation of cell proliferation in midbrain; negative regulation of epithelial cell proliferation; negative regulation of fat cell differentiation; negative regulation of fibroblast growth factor receptor signaling pathway; negative regulation of melanin biosynthetic process; negative regulation of mesenchymal cell proliferation; negative regulation of prostatic bud formation; negative regulation of synapse assembly; negative regulation of transcription, DNA-templated; neuron differentiation; non-canonical Wnt signaling pathway; non-canonical Wnt signaling pathway via JNK cascade; notochord morphogenesis; olfactory bulb interneuron development; optic cup formation involved in camera-type eye development; paraxial mesoderm formation; planar cell polarity pathway involved in axis elongation; planar cell polarity pathway involved in axon guidance; planar cell polarity pathway involved in cardiac muscle tissue morphogenesis; planar cell polarity pathway involved in cardiac right atrium morphogenesis; planar cell polarity pathway involved in gastrula mediolateral intercalation; planar cell polarity pathway involved in midbrain dopaminergic neuron differentiation; planar cell polarity pathway involved in neural tube closure; planar cell polarity pathway involved in outflow tract morphogenesis; planar cell polarity pathway involved in pericardium morphogenesis; positive regulation of angiogenesis; positive regulation of cartilage development; positive regulation of cell–cell adhesion mediated by cadherin; positive regulation of chemokine biosynthetic process; positive regulation of cytokine production involved in immune response; positive regulation of endocytosis; positive regulation of endothelial cell migration; positive regulation of endothelial cell proliferation; positive regulation of fibroblast proliferation; positive regulation of heart induction by negative regulation of canonical Wnt signaling pathway; positive regulation of inflammatory response; positive regulation of interferon-gamma production; positive regulation of interleukin-1 beta production; positive regulation of interleukin-6 production; positive regulation of interleukin-8 production; positive regulation of macrophage activation; positive regulation of macrophage cytokine production; positive regulation of meiotic nuclear division; positive regulation of mesenchymal cell proliferation; positive regulation of neuron death; positive regulation of neuron projection arborization; positive regulation of neuron projection development; positive regulation of NF-KappaB transcription factor activity; positive regulation of non-canonical Wnt signaling pathway; positive regulation of ossification; positive regulation of peptidyl-serine phosphorylation; positive regulation of peptidyl-threonine phosphorylation; positive regulation of protein binding; positive regulation of protein catabolic process; positive regulation of protein kinase C activity; positive regulation of protein kinase C signaling; positive regulation of protein localization to synapse; positive regulation of response to cytokine stimulus; positive regulation of T cell chemotaxis; positive regulation of thymocyte apoptotic process; positive regulation of timing of anagen; positive regulation of transcription, DNA-templated; positive regulation of transcription by RNA polymerase II; positive regulation of tumor necrosis factor secretion; positive regulation of type I interferon-mediated signaling pathway; post-anal tail morphogenesis; postsynapse assembly; presynapse assembly; primary heart field specification; primitive streak formation; regulation of branching involved in mammary gland duct morphogenesis; regulation of cellular protein localization; regulation of I-KappaB kinase/NF-KappaB signaling; regulation of inflammatory response; regulation of postsynapse organization; regulation of postsynaptic cytosolic calcium ion concentration; response to organic substance; secondary heart field specification; secondary palate development; somitogenesis; type B pancreatic cell development; urinary bladder development; uterus development; vagina development; Wnt signaling pathway; Wnt signaling pathway, calcium modulating pathway; Wnt signaling pathway, planar cell polarity pathway; Wnt signaling pathway involved in midbrain dopaminergic neuron differentiation; wound healing
RARA	Retinoic acid receptor alpha	Nucleus	apoptotic cell clearance; cell differentiation; cellular response to estrogen stimulus; cellular response to lipopolysaccharide; chondroblast differentiation; embryonic camera-type eye development; face development; female pregnancy; germ cell development; glandular epithelial cell development; growth plate cartilage development; hippocampus development; hormone-mediated signaling pathway; limb development; liver development; multicellular organism growth; negative regulation of apoptotic process; negative regulation of cartilage development; negative regulation of cell population proliferation; negative regulation of granulocyte differentiation; negative regulation of interferon-gamma production; negative regulation of transcription, DNA-templated; negative regulation of transcription by RNA polymerase II; negative regulation of tumor necrosis factor production; neural tube closure; outflow tract septum morphogenesis; positive regulation of binding; positive regulation of cell cycle; positive regulation of cell population proliferation; positive regulation of interleukin-13 production; positive regulation of interleukin-4 production; positive regulation of interleukin-5 production; positive regulation of neuron differentiation; positive regulation of T helper 2 cell differentiation; positive regulation of transcription, DNA templated; positive regulation of transcription by RNA polymerase II; prostate gland development; protein phosphorylation; regulation of myelination; regulation of synaptic plasticity; response to cytokine; response to estradiol; response to ethanol; response to retinoic acid; response to vitamin A; retinoic acid receptor signaling pathway; Sertoli cell fate commitment; signal transduction; spermatogenesis; trachea cartilage development; transcription initiation from RNA polymerase II promoter; ureteric bud development; ventricular cardiac muscle cell differentiation
SULF1	Extracellular sulfatase Sulf-1	Endoplasmic reticulum; Golgi apparatus	apoptotic process; bone development; cartilage development; chondrocyte development; embryonic skeletal system development; esophagus smooth muscle contraction; glial cell-derived neurotrophic factor receptor signaling pathway; glomerular basement membrane development; glomerular filtration; heparan sulfate proteoglycan metabolic process; innervation; kidney development; negative regulation of angiogenesis; negative regulation of cell migration; negative regulation of endothelial cell proliferation; negative regulation of fibroblast growth factor receptor signaling pathway; negative regulation of prostatic bud formation; positive regulation of BMP signaling pathway; positive regulation of vascular endothelial growth factor production; positive regulation of Wnt signaling pathway; regulation of fibroblast growth factor receptor signaling pathway; vascular endothelial growth factor receptor signaling pathway

**Table 4:** Genes with different multiples of change in females and males.

Symbol	Gene Name	Male			Female		Male vs. Female	
		OA vs. Healthy			OA vs. Healthy			
		Log_2_FC	*p* Value	Log_2_FC		*p* Value	Log_2_FC	*p* Value
COL1A1	collagen type I alpha 1 chain	3.791243	3.33 × 10^−4^	7.250090		8.76 × 10^−7^	−3.458847	3.88 × 10^−2^
COL1A2	collagen type I alpha 2 chain	2.206448	2.20 × 10^−2^	5.722798		2.93 × 10^−8^	−3.516351	4.58 × 10^−3^
MXRA5	matrix remodeling associated 5	2.179711	4.13 × 10^−2^	5.721745		4.35 × 10^−6^	−3.542034	1.59 × 10^−2^
THY1	Thy-1 cell surface antigen	3.064184	3.65 × 10^−4^	7.253767		9.26 × 10^−7^	−4.189584	7.67 × 10^−3^
TNFAIP6	TNF alpha induced protein 6	3.028402	1.71 × 10^−3^	7.264821		8.86 × 10^−6^	−4.236418	1.56 × 10^−2^

## Data Availability

The data presented in this study are contained within this article and Supplementary materials.

## Supplementary Materials

Supplementary materials can be found at https://www.mdpi.com/1422-0067/22/2/569/s1. Table S1. The information of the samples included in the current study. Table S2. The list of the identified genes statistically significantly changed in OA cartilage of males. Table S3. The list of the identified genes statistically significantly changed in OA cartilage of females. Table S4. The list of the identified genes statistically significantly upregulated in OA cartilage of both genders. Table S5. The list of the identified genes statistically significantly downregulated in OA cartilage of both genders. Table S6. The list of the identified genes statistically significantly different between genders.
